# Milk Supply in Lebanon: Economic Challenges and the Role of Traditional Dairy Products

**DOI:** 10.3390/foods14173115

**Published:** 2025-09-05

**Authors:** Ossama Dimassi, Lina Jaber, Layla Fleyfel, Shady Hamadeh

**Affiliations:** 1 Department of Research, American University of Europe, New York, NY 10017, USA; 2 Environment and Sustainable Development Unit, Faculty of Agricultural and Food Sciences, American University of Beirut, Beirut P.O. Box 11-236, Lebanon; lj01@aub.edu.lb (L.J.); lf41@aub.edu.lb (L.F.); shamadeh@aub.edu.lb (S.H.); 3 Department of Agriculture, Faculty of Agricultural and Food Sciences, American University of Beirut, Beirut P.O. Box 11-236, Lebanon

**Keywords:** traditional dairy products, Lebanon, Labneh, Halloumi, Shanklish, Kishk, whey valorization, sustainable food systems, cheese classification, circular economy

## Abstract

Traditional dairy products remain an essential yet underutilized component of Lebanon’s food system. Amid economic instability, supply chain fragility, and heavy reliance on imported dairy inputs (≈80% of demand), these products offer resilient, low-input alternatives rooted in centuries-old practices. This review analyzes key traditional Lebanese dairy products, including Labneh, Labneh–Anbaris, Akkawi, Shanklish, Halloumi, Karishi, Pressed–Brined Karishi (Lebanese Double-Cream), Qishta, and Kishk, using Codex Alimentarius and Tetra Pak classification frameworks. It examines their compositional attributes, milk-to-product conversion efficiency, preservation methods, and economic characteristics. The findings reveal a continuum from high-yield fresh cheeses to lower-yield preserved forms with extended shelf life, demonstrating diversified strategies for food security and resilience. Unlike prior studies focused mainly on composition or culinary aspects, this review integrates classification systems with cultural geography to map Lebanon’s traditional dairy landscape. It highlights strategies grounded in rural milk availability and artisanal know-how, revealing overlooked food system functions. These practices exemplify circular models that valorize whey, minimize waste, and preserve quality without refrigeration, aligning with sustainability goal SDG-12.3. This review calls for integrating these products into national food strategies, regulatory frameworks, and innovation systems, recognizing traditional Lebanese dairy as both cultural heritage and a strategic resource for a more self-sufficient and resilient food sector.

## 1. Introduction

The dairy industry in Lebanon is a cornerstone of the country’s agricultural sector, contributing to essential nutrition, rural livelihoods, and the preservation of cultural heritage. However, the sector faces significant challenges, including a heavy reliance on imported milk, high production costs, and ongoing political instability. These factors have hindered the growth of local production and threatened the sustainability of the industry. Despite these difficulties, Lebanon’s rich tradition of artisanal dairy products, such as Labneh, Labneh Anbaris, Shanklish, Akkawi, Halloumi, Karishi, Qishta, and Kishk, offers unique opportunities for economic growth and market competitiveness.

This review critically examines the current state of milk supply in Lebanon, with particular emphasis on the challenges, opportunities, and potential associated with traditional dairy products and cheese varieties. By addressing key research questions, this paper seeks to generate evidence-based insights that can inform strategies for the sustainable development of the Lebanese dairy sector.

### 1.1. Key Research Questions

As Lebanon continues to navigate a complex landscape of challenges and opportunities within its dairy industry, several key research questions arise:

- What are the main challenges facing Lebanon’s milk supply, including local production constraints and reliance on imports?
- How can Lebanon leverage its traditional dairy products, such as Labneh, Labneh Anbaris, Shanklish, Halloum, Karishi, Pressed–Brined Karishi (locally called Double Cream), Qishta, and Kishk, to enhance market competitiveness and profitability?
- How can innovation in cheese-making, sustainable practices, and modern technology drive Lebanon’s dairy industry forward and reduce its reliance on imported milk?

According to FAOSTAT trade data [1], Lebanon continues to exhibit a sustained structural dependency on imported dairy commodities. Between 2019 and 2023, the country imported an annual average of approximately 20,000 tonnes of cheese produced from whole cow’s milk, while exports remained below 500 tonnes—resulting in a trade deficit exceeding 95%. These figures are corroborated by domestic assessments, such as the Blominvest Bank report (2023) [2], which confirms that despite significant seasonal milk production capacity, Lebanon’s dairy sector remains heavily import-dependent.

Moreover, annual imports of whole milk powder and skim milk powder averaged 2100 tonnes and 1500 tonnes, respectively, underscoring the widespread use of reconstituted milk in domestic processing. These patterns persisted amid global dairy price volatility and local currency depreciation, highlighting the inherent fragility of the current supply model. Additionally, Lebanon imported notable volumes of processed dairy products not elsewhere classified, suggesting growing reliance on industrial dairy inputs. Collectively, these trends underscore the urgent need to reinvest in locally embedded, seasonally responsive dairy systems that are less vulnerable to international market fluctuations.

In response to these challenges, the present review examines the classification, production efficiency, and policy relevance of Lebanon’s traditional dairy sector. Addressing the identified research questions is critical for achieving the sustainable growth of the dairy industry, with the broader goals of restoring self-sufficiency, promoting innovation, and generating new economic opportunities.

This review offers evidence-based insights to inform targeted interventions that strengthen local milk production, reduce import dependence, and unlock the economic value of traditional dairy products. By focusing on innovation, sustainability, and strategic market development, this study outlines a pathway through which Lebanon may position itself as a more resilient and competitive actor in both regional and global dairy markets. In doing so, the country may advance toward a more self-reliant and sustainable dairy sector capable of preserving cultural heritage while responding to modern economic and environmental imperatives.

### 1.2. Contextualizing Lebanon’s Dairy Industry

Lebanon’s dairy industry is deeply rooted in the country’s agricultural and cultural history. For centuries, dairy farming has been an integral part of rural life, providing sustenance and economic stability for many communities [3,4,5]. The country’s mountainous terrain and temperate climate have historically been conducive to animal husbandry, particularly for sheep, goats, and cows, which have been the primary sources of milk production. This fertile land, coupled with traditional knowledge passed down through generations, has shaped Lebanon’s dairy sector into one that offers a rich array of products that are not only staples in the Lebanese diet but also central to the country’s culinary identity [4,5,6,7,8,9,10].

At the heart of Lebanon’s dairy history is the production of traditional dairy products, often crafted in small family-run farms and cooperatives, reflecting the country’s rich heritage of artisanal cheese-making [4,9]. Traditional dairy products, such as Labneh, Labneh Anbaris, Shanklish, Halloumi, and Kishk, are not only staples in the Lebanese diet but also central to the country’s culinary identity [3,7]. These locally made products represent both a vital cultural heritage and an essential part of the Lebanese economy [3,6,7,11]. Over time, as the global market evolved and technology advanced, Lebanon’s dairy industry witnessed the rise of large-scale production; however, traditional methods have continued to hold cultural and economic significance [12]. Despite these developments, Lebanon’s dairy industry faces a number of challenges, particularly regarding self-sufficiency, reliance on imports, and the evolving demands of both local and global markets.

## 2. The Role of Traditional Dairy Products in Lebanese Cuisine

Lebanon’s traditional dairy products represent a dynamic interplay between ecological adaptation, cultural transmission, and technical ingenuity. Produced predominantly at the household or village cooperative level, these products are not only daily staples but also emblematic of regional identity and intergenerational know-how. Their production techniques vary widely according to geography, seasonality, and the available milk types, with many items manifesting both fresh and mature forms, each with distinct textures, flavors, and culinary applications. The following overview outlines the major products, emphasizing their preparation modes, consumption patterns, and cultural context.

### 2.1. Labneh

Labneh (لبنة) is a strained yogurt product that holds a central place in the Lebanese diet. Typically consumed fresh, Labneh is produced by draining yogurt through cloth, resulting in a thick, spreadable paste characterized by tangy and mildly acidic notes. It is commonly served as part of daily breakfasts or mezze platters, often accompanied by olive oil and herbs.

A mature variant of Labneh, known as Labneh makbouseh, involves rolling the strained product into balls and preserving them in olive oil. This method extends shelf life and produces a more concentrated flavor. The contrast between the fresh and preserved forms of Labneh illustrates its dual function as both a perishable dairy item and a long-term storage food, particularly in households prior to the widespread use of refrigeration [2,13,14,15,16].

In mountainous regions such as the Chouf and areas of the Bekaa Valley, Labneh makbouseh is traditionally prepared at the end of the spring milking season as a strategy for dairy preservation. Although direct ethnographic documentation is limited, this practice is consistent with the logic of seasonal surplus management and provisioning in Lebanon’s agropastoral communities, as discussed by Hosri (2016) [17] and Kaaki (2012) [15].

### 2.2. Labneh Anbaris

Labneh Anbaris (لبنة قنبريس) is a traditional fermented dairy product derived from Labneh, characterized by extended fermentation and aging in sealed earthenware vessels known as anbaris. This process typically lasts several months and results in a sharp, dense, and slightly crumbly product. In contrast to fresh Labneh, which is mildly tangy and intended for immediate consumption, Labneh Anbaris is markedly pungent and acidic, with properties designed for long-term storage.

Its production is seasonal and aligned with the spring milking period, when milk is abundant. This timing reflects its role in traditional food preservation systems in Lebanon’s mountain communities. The Chouf and western Bekaa regions are particularly known for their production of Labneh Anbaris, where it is prepared during the spring and stored in clay containers for winter use [3,18].

The mature profile of Anbaris represents a historical adaptation to environments with limited access to refrigeration, reflecting the agrarian need for shelf-stable, nutrient-dense dairy reserves [3,18,19]. In some cases, the aged Labneh is shaped into small balls and preserved in olive oil, which not only prolongs its shelf life but also imparts herbal and aromatic notes. In this form, Labneh Anbaris shares similarities with Labneh makbouseh and aligns with comparable standards in terms of composition and classification.

The rising global interest in fermented and probiotic-rich foods positions Labneh Anbaris as both a culturally significant and nutritionally valuable product. Its distinctive sensory attributes and artisanal character underscore its potential for broader international recognition and niche market development [20,21].

### 2.3. Shanklish

Shanklish (شنكليش) is a mold-ripened, aged cheese produced by fermenting yogurt curd, shaping it into balls, and subjecting it to sun-drying followed by ambient aging. It is typically coated with thyme (za’atar), sumac, or chili powder to enhance its flavor and promote surface microbial development. Mature Shanklish is aged for several months, resulting in a pungent aroma, brittle texture, and pronounced flavor. In contrast, a fresher version, locally referred to as “akhdar” or green Shanklish, is consumed within days of shaping and offers a milder taste and softer texture [22].

The mature form, often stored for approximately 30 days in a dark, sealed container, is usually crumbled into salads. The fresh variant is commonly sliced and served with tomatoes and onions. These two degrees of ripeness correspond to regional and generational preferences. In northern Lebanon, drier and more intensely ripened forms are generally favored, while in southern areas, Shanklish is often consumed earlier in the aging cycle [20,23,24].

The village of Rahbeh in North Lebanon is especially known for its distinctive cave-aged Shanklish, which is traditionally prepared during summer and consumed in winter. This traditional product holds symbolic significance in Levantine food culture and has been identified as a potential candidate for Protected Geographical Indication (PGI) recognition [24]. Its production serves as both an effective method of milk preservation and a culturally rooted strategy for reducing seasonal surplus in small-scale farming systems.

### 2.4. Akkawi

Akkawi (عكاوي) is a soft white cheese preserved in brine and widely consumed across Lebanon and the broader Levant. Although its origin is linked to the city of Acre in historical Palestine [25], Akkawi has become a foundational component of Lebanese dairy traditions. It is most commonly produced from cow’s milk, although artisanal versions prepared from sheep or goat milk are still encountered in rural contexts.

Akkawi is available in both fresh and aged forms. Fresh Akkawi is mildly salty, elastic, and uniform in texture. It is typically eaten with bread or incorporated into pastries and desserts such as kunafa, where its smooth melting properties are especially valued. The aged or pickled variant, sometimes referred to locally as Akkawi Tscheeky, is preserved in brine for extended periods. This process yields a firmer texture and a more concentrated flavor. Historically, this preservation practice allowed for the continuous availability of dairy products throughout the year and reflects broader strategies of food conservation employed across the Eastern Mediterranean [26,27,28]. The name Tscheeky originates from its production in the Czech Republic by Lebanese traders for the purpose of export to Lebanon and the Arab world [28].

Akkawi occupies an important place in both daily and ceremonial food practices. It is consumed as a table cheese, grated into cooked dishes, or layered in oven-baked specialties. This culinary adaptability has contributed to its enduring popularity across generations. The distinction between its fresh and aged forms reflects not only sensory preferences but also differences in household storage capacity and regional food customs. As such, Akkawi serves both as a fresh dairy item and as a preserved protein source within traditional Lebanese food systems.

Although Akkawi is available throughout Lebanon, its production is particularly concentrated in the Bekaa Valley and the southern coastal regions, including Chtoura and Saida [29]. The production of its fresh, brined form coincides with seasonal milk surpluses during early summer, especially from small ruminants raised in pastoral systems [30]. Akkawi also serves as a foundational curd for other traditional cheeses, including Karishi and Pressed–Brined Karishi, reinforcing its functional importance within the local dairy economy.

### 2.5. Halloumi

Halloumi (حلوم) is a semi-hard cheese preserved in brine and traditionally produced from sheep’s milk, although contemporary versions incorporating mixed or cow’s milk are increasingly common. It is consumed in both fresh and aged forms. The term “Halloumi” is linguistically derived from the Arabic words ḥallam or ḥalīm, which connote characteristics of firmness and grace, reflecting the cheese’s ability to retain its structure under heat while maintaining a supple texture.

Fresh Halloumi is white, pliable, and mildly salty, making it particularly suitable for grilling or pan-frying because of its high melting point. When stored in brine, Halloumi gradually becomes saltier and firmer in texture. In some contexts, the cheese is intentionally aged for several weeks or months, after which it may be grated or incorporated into cooked dishes. The distinction between its fresh and aged forms is influenced by local storage conditions and culinary applications, ranging from its use as a table cheese to a preserved ingredient in pies or stews [31,32,33].

While Halloumi is widely consumed in Lebanon, it has also gained international significance following the successful registration of the term as a Protected Designation of Origin (PDO) by the Republic of Cyprus in 2021 [34]. This legal protection restricts the use of the name “Halloumi” to cheese produced in Cyprus under defined production conditions, including the requirement that no less than 51 percent of the milk used must originate from sheep or goats.

In Lebanon, Halloumi is traditionally produced in various regions, with particular documentation from the Bekaa Valley and South Lebanon, including towns such as Chtoura and Saida. Production is typically concentrated in late spring and early summer, coinciding with the seasonal abundance of sheep and goat milk following the post-lambing period [12]. The characteristic folding and boiling method used in Halloumi preparation reflects Levantine pastoral heritage and has been preserved through artisanal knowledge transmitted across generations [35]. Historical records from the FAO indicate that Lebanese Halloum was traditionally shaped into round forms and aged briefly prior to cooking, although this artisanal practice has increasingly been supplanted by industrial processing methods utilizing pasteurized milk [35].

### 2.6. Karishi

Karishi (قريشة), also known locally as Areesheh, is a traditional Lebanese whey cheese produced by reheating the residual whey obtained following the preparation of curd or Halloumi. It is a fresh, soft cheese characterized by a delicate, slightly sweet flavor and a moist, crumbly texture. Often referred to as Lebanese ricotta, Karishi is typically prepared in small batches at the household or village level, reflecting a resource-efficient approach to dairy processing that enhances the nutritional and economic value of byproducts derived from cheese-making. Due to its high moisture content and the absence of preservatives, Karishi is consumed within a few days of production and does not traditionally exist in an aged or preserved form [31,36].

Culturally, Karishi is most often served at breakfast, accompanied by fresh bread, tomatoes, or olive oil, and is also used in traditional desserts and stuffed pastries. Although it is usually unsalted or only lightly salted, regional and household variations may include firmer, more drained forms that allow for modestly extended storage. This cheese is traditionally produced in rural regions of Lebanon and has been particularly documented in the Bekaa Valley, where it is made during spring and early summer, following peak Halloumi production [12,24,30].

Karishi exemplifies traditional preservation methods embedded in seasonal dairy sequencing and reflects a broader commitment to minimizing waste in artisanal food systems [37]. Its continued presence in home kitchens and small dairies attests to its cultural significance as a representation of frugality, freshness, and stewardship within Lebanon’s dairy heritage. The seasonal pattern of Karishi production parallels that of Halloumi and Akkawi, which are also concentrated in late spring and early summer, when milk volumes are at their highest. This seasonal rhythm is especially well documented in areas such as the Bekaa Valley and Mount Lebanon [17,30,35].

Karishi also serves as the base ingredient for Lebanese Double-Cream cheese, a product that resembles ricotta salata in its composition and culinary application [38], as discussed in the following section.

### 2.7. Lebanese Double-Cream (Pressed–Brined Karishi)

Lebanese Double-Cream, although suggestive of a high-fat dairy product, is not a true cream but rather a traditional cheese derived from Karishi. It is produced by salting and pressing Karishi curds to expel residual whey, resulting in a denser, firmer, and more cohesive texture. This cheese is typically prepared in rural areas of the Bekaa Valley during the spring and early summer months, when Karishi is readily available following peak Halloumi production. Its preparation illustrates preservation strategies grounded in the efficient use of seasonal surpluses and minimal waste [37].

The final product is usually shaped into blocks or patties and consumed fresh or within a limited storage period. Its mild flavor and compact structure make it suitable for consumption with bread or vegetables in both rural and urban households.

Although colloquially referred to as “Double-Cream”, the product does not contain added fat or cream, distinguishing it from commercial dairy spreads and high-fat creams. Instead, its richness is attributed to the concentration of curd solids achieved through pressing. In some local contexts, salt-curing is employed to extend shelf life slightly beyond that of fresh Karishi, though the product remains fundamentally fresh in nature.

Lebanese Double-Cream reflects the traditional use of whey-derived curds within Lebanese dairy practices and highlights a continued emphasis on low-waste, small-scale dairy processing [39]. Its production parallels that of ricotta salata, in which fresh whey cheeses are pressed and salted to produce a firmer, more transportable version with enhanced economic and culinary value [38].

### 2.8. Qishta

Qishta (قشطة) is a traditional Lebanese dairy product produced through the controlled heating and skimming of whole milk in wide, shallow, open vessels. Due to its method of formation, it is often described as a collected milk skin [40]. The origins of Qishta production in Lebanon are culturally embedded and historically associated with rural farmhouse practices, where it served as a method for preserving surplus milk. Over time, Qishta has evolved from a subsistence dairy item into a central component of Lebanese confectionery and pastry-making. It is now a key filling in iconic desserts such as Knefe, Mafrouke, and Atayef [6,41].

Although the specific regional origins of Qishta are not clearly documented in the existing literature, its preparation is widespread throughout Lebanon and retains numerous artisanal characteristics [42]. The traditional method involves the slow heating of milk in large, shallow pans without mechanical agitation, allowing for the natural formation and manual collection of a creamy surface layer. This process preserves the product’s soft texture and distinctive layered appearance [6,43]. Qishta is not typically consumed on its own but is valued for its rich, stable texture and mild dairy flavor, which maintains integrity under baking conditions.

In recent decades, the production of Qishta has undergone a degree of commercialization, particularly in northern Lebanese cities such as Tripoli. Notably, established confectionery manufacturers such as Hallab 1881 have adopted traditional production principles while employing modified equipment to enable larger-scale output without compromising artisanal quality [6]. Despite these developments, many small workshops and households continue to use the conventional shallow-pan method. Due to fluctuations in fresh milk availability, some producers now supplement whole milk with reconstituted milk powder to maintain consistent yields [6,41,43].

The role of Qishta in Lebanese culinary heritage highlights the adaptation of heat treatment technologies to local contexts and sensory expectations, transforming highly perishable raw milk into a versatile, semi-dehydrated, high-value ingredient. The process incorporates traditional knowledge of protein denaturation, fat coalescence, and water evaporation, resulting in a product that remains distinctly Lebanese. Although related to dairy preparations such as Turkish Kaymak and Indian Khoa, Qishta retains a unique identity rooted in Lebanese foodways [6,41]. As consumer demand for traditional desserts persists in both domestic and diaspora markets, Qishta production continues to balance artisanal craftsmanship with opportunities for scalable and standardized processing.

### 2.9. Kishk

Kishk (كشك) is a traditional Lebanese fermented dairy and cereal product prepared by blending yogurt and/or Labneh with burghul (parboiled, cracked, and dried wheat), followed by an extended period of fermentation and drying. The resulting dried mixture is then ground into a fine, shelf-stable powder that can be stored for long durations without refrigeration. While a soft intermediate variant known as Kishk akhdar may be consumed during production, the dried powder represents the standard form. It is commonly used in soups, stews, and porridges, particularly during the winter months. Kishk is noted for its high nutritional density, lactic acid content, and probiotic activity, all of which contribute to both its distinctive flavor and its digestive benefits [7,36,44,45,46].

The product is traditionally prepared in late summer across several regions of Lebanon, including North Bekaa, West Bekaa, the Chouf, and Akkar. In these areas, yogurt and bulgur are sun-dried on rooftops as part of seasonal food preparation cycles. As a fermented Mouneh staple, Kishk is valued for its extended shelf life and is deeply embedded in local shepherding traditions and agropastoral knowledge systems [24,45].

Kishk exemplifies Lebanon’s adaptive food preservation strategies, particularly in mountainous and semi-arid regions characterized by seasonal milk surpluses. Its production extends the utility of dairy resources by integrating protein and cereal into a compact and transportable format. The process represents a resource-efficient model in which fermentation and dehydration transform perishable ingredients into durable nutritional staples. Artisanal Kishk production continues in rural households and village cooperatives, where inherited techniques preserve its cultural and economic relevance.

More broadly, traditional dairy products occupy an essential role in Lebanese culinary identity. They contribute not only to the flavor profile of everyday and festive meals but also serve as markers of hospitality and communal practice. These products offer economically viable options for smallholder farmers by reducing post-harvest losses and transforming surplus milk into value-added goods. Drawing on Lebanon’s culinary heritage, traditional dairy products provide a distinctive competitive advantage in both domestic and export markets by aligning artisanal quality with cultural authenticity.

## 3. Challenges Facing Lebanon’s Dairy Industry

### 3.1. Economic Pressures

Traditional dairy producers in Lebanon operate under mounting economic pressure. The cost of animal feed has increased significantly due to inflation and currency devaluation, especially in the case of imported concentrates and grains that are essential for maintaining consistent milk yields [10,17]. Energy expenses have escalated following the collapse of the national power grid, forcing producers to rely on diesel generators for milk cooling, pasteurization, and storage. These expenditures are frequently beyond the financial capacity of small-scale producers [10,30]. Labor shortages and rising wages in both rural and urban areas further constrain operations, particularly for labor-intensive products such as Shanklish and Labneh Anbaris.

Additional costs related to packaging and veterinary services have also risen, while imported equipment used in cheese production and fermentation is increasingly inaccessible due to banking restrictions and the shortage of foreign currency. In contrast to producers in countries such as Cyprus or Greece, Lebanese dairy farmers generally lack cooperative frameworks that could buffer input prices or support bulk purchasing strategies [47]. In the absence of targeted subsidies or technical assistance, the cost structure of artisanal dairy production remains highly vulnerable.

### 3.2. Political Instability and Infrastructure Gaps

Lebanon’s ongoing political and institutional instability has significantly disrupted dairy production, particularly across rural regions. Frequent electricity outages, irregular water supply, and deteriorating public services have impaired all stages of the dairy value chain, including milking, cooling, transportation, and retail distribution [17]. Public veterinary services and food safety oversight have been weakened by administrative paralysis, resource constraints, and the departure of trained personnel [10,48]. This erosion of state capacity has contributed to the expansion of informal dairy processing, often in the absence of regulatory support or microbiological monitoring.

Infrastructural decline has further marginalized mountain and peri-urban producers, limiting their access to regional markets. Poor road networks, insufficient transport capacity, and rising fuel prices delay milk collection and constrain the timely delivery of perishable products. Garçon and Zurayk (2011) [12] emphasized that the state’s retreat from food systems planning has hindered the integration of culturally significant traditional products into formal commercial channels. In addition, the World Bank’s agricultural sector assessment highlights how chronic underinvestment in rural infrastructure and the absence of a national strategy for including smallholders in formal value chains have undermined both agricultural productivity and employment generation in the sector [49]. As a result, artisanal dairy producers remain structurally excluded from high-value markets, despite increasing domestic and international demand for traditional Lebanese dairy products.

### 3.3. Import Dependency and Market Volatility

Lebanon’s dairy sector remains heavily dependent on international markets for both finished products and essential production inputs. The country currently imports between seventy and eighty percent of its total dairy consumption, including powdered milk, industrial cheeses, and packaged dairy goods [2,10]. Local production also relies on imported fuels, fermentation enzymes, packaging materials, and equipment used in cheese processing [17]. In light of the collapse of the national currency and ongoing banking restrictions, many of these imports have become unaffordable for producers operating in a devalued local currency. As a result, profit margins have declined and investment in dairy infrastructure has significantly slowed.

The volatility of global trade and energy markets has added further instability to the sector. Fluctuations in fuel prices and shipping disruptions have substantially increased freight costs and contributed to interruptions in cold chain logistics, particularly for imported goods [10]. During port closures and periods of economic disruption, the price of milk powder has risen sharply, exposing the inherent fragility of Lebanon’s dependence on external suppliers [2]. Dubeuf et al. (2016) [30] observed that such vulnerabilities are common across Mediterranean small ruminant systems, although Lebanon’s challenges are exacerbated by the absence of coordinated food security planning. The World Bank has similarly attributed Lebanon’s continued reliance on dairy imports to structural governance constraints and insufficient investment in domestic livestock value chains [49].

Within this context, the expansion of traditional dairy processing may serve as a strategic buffer. Many artisanal products, such as Kishk, Anbaris, and Shanklish, require minimal imported inputs, are suitable for storage without refrigeration, and can act as substitutes during periods of supply chain disruption. These same products also hold potential for niche export markets, particularly among diaspora communities seeking culturally significant and traditionally prepared foods.

### 3.4. Food Safety Risks and Limitations of Traditional Production

Although traditional Lebanese dairy products are valued for their extended shelf stability and cultural resilience, the artisanal production methods by which they are prepared present several food safety concerns. In many cases, hygienic practices remain informal and are not subject to regulatory oversight, particularly among small-scale producers. For instance, Kishk and Shanklish are commonly sun-dried and aged, respectively, in open environments without microbial control, creating favorable conditions for contamination by spoilage organisms or pathogenic bacteria [50,51].

Anbaris production depends on spontaneous fermentation facilitated by ambient microbial flora and is not guided by defined starter cultures. This approach results in variable acidification rates and uncertain inhibition of pathogenic microorganisms [3,18]. In a related example, Qishta is prepared by coagulating milk on the surface of open shallow pans. If the heating temperature is suboptimal or cooling is delayed, the product becomes highly susceptible to microbial spoilage due to its elevated moisture content and limited shelf life [42].

These microbiological risks are further compounded by infrastructural constraints such as inadequate access to stainless steel processing equipment, reliable refrigeration, and potable water in rural dairy facilities [17]. Strengthening microbial monitoring and incorporating affordable sanitary interventions into traditional workflows are, therefore, essential steps in ensuring the safe scaling of artisanal dairy production.

These challenges are consistent with the findings obtained by Abebe et al. (2017) [52], whose analysis of governance mechanisms in the Lebanese dairy sector revealed that only 53 percent of commercial farms received food safety inspections. Most producers operated under informal verbal agreements and lacked standardized hygiene protocols. This reflects a broader institutional deficiency in the enforcement of on-farm food safety practices, one that cannot be sustainably resolved through Food Safety Management Systems (FSMSs) alone in the absence of integrated regulatory support and consistent monitoring frameworks.

## 4. Technical Classification, Composition, and Milk Conversion Efficiency of Traditional Dairy Products

Understanding milk-to-dairy product conversion values is essential for the economic evaluation of traditional cheese production. These values directly influence yield estimation, raw material costs, and pricing strategies. Traditional dairy processing methods are generally less efficient than industrial techniques, often requiring a greater volume of milk to produce a given quantity of cheese. This inefficiency becomes especially important in regions with fluctuating milk prices or limited supply, where production costs are tightly linked to conversion ratios.

The classification of cheese, as outlined in Table 1, adopted from Tetra Pak Dairy Processing Handbook (2015) [53], provides a framework for understanding these conversion values in relation to key compositional and physical characteristics. This classification uses parameters such as Moisture on a Fat-Free Basis (MFFB), Fat in Dry Substance (FDS), fat level, texture or hardness, and ripening status to categorize cheeses into types such as extra-hard, hard, semi-hard, semi-soft, and soft. For instance, extra-hard cheeses have an MFFB of less than 41% and are typically cured or ripened, while soft cheeses have an MFFB greater than 67% and are usually uncured or fresh. The level of fat, ranging from high fat (>60% FDS) to skim (<10% FDS), also influences both classification and yield.

These compositional differences have significant implications for milk-to-cheese conversion. Cheeses with a lower moisture content and longer ripening times, such as extra-hard or hard cheeses, require a greater quantity of milk to produce one kilogram of finished product. On the other hand, soft, fresh cheeses retain more moisture and experience minimal shrinkage during processing, resulting in higher yields. Semi-hard and semi-soft cheeses, which fall within intermediate MFFB and FDS ranges, tend to yield moderate amounts and may be either surface- or interior-ripened.

In addition to the cheese type, the source of milk (cow, goat, or sheep) further affects conversion values due to variations in protein, fat, and total solids. A higher solids content in sheep milk, for example, often leads to higher cheese yields, particularly in the production of hard and semi-hard varieties. Regulatory requirements concerning minimum fat, protein, and solids-not-fat levels in the final product also influence the quantity of milk required, as additional milk or solids may be needed to meet quality standards.

By integrating precise milk-to-cheese conversion ratios within a standardized classification framework, such as that in Table 1, dairy producers can achieve more accurate cost assessments, better resource planning, and improved production efficiency. This is especially critical in traditional cheese manufacturing, where consistency, economic sustainability, and regulatory compliance must all be balanced to ensure long-term viability.

### 4.1. Labneh

#### 4.1.1. Fresh Labneh

Fresh Labneh is ready for consumption immediately after the straining process and represents the most common form consumed daily. Traditional Lebanese Labneh is produced by lactic acid fermentation of milk, followed by removal of whey to achieve a thick, spreadable consistency. It is typically prepared by draining yogurt through cloth bags. In the industrial process, ultrafiltration is used. This method concentrates milk solids while preserving the lactic acid bacteria responsible for its distinctive tangy flavor and probiotic benefits [54,55].

Composition: Fresh Labneh typically contains approximately 9.4% fat, 7.7% protein, and 72.5% moisture, with a pH of 4.2 and titratable acidity measured at 1.02% lactic acid [44,56].

Kg milk to kg Labneh: Traditional production requires approximately 2.6 kg of 4% fat cow milk per 1 kg of Labneh, while ultrafiltration methods may reduce this to 2.0 kg/kg [55]. Goat milk offers comparable yields, whereas sheep milk, with a naturally higher fat content, may be used to achieve richer Labneh with less total milk input [25,56].

Shelf Life: Fresh Labneh is highly perishable, with a typical refrigerated shelf life of 10–15 days at 4 °C. Its spoilage is primarily driven by continued acidification and potential fungal growth, which may be mitigated by refrigeration or the use of permitted preservatives in commercial products [56,57,58].

Classification: According to the Tetra Pak Cheese Classification Matrix (2015) and Codex Alimentarius CXS 243-2003 [53,59], fresh Labneh is an “unripened fresh cheese” with a high MFFB (>67%) and a low- to medium-fat FDS content. It retains active cultures without any ripening phase, placing it firmly in the fresh cheese category suitable for short-term consumption.

#### 4.1.2. Labneh Balls

Definition: Labneh Balls (لبنة مكبوسة) represent a matured form of Lebanese Labneh. This variant is prepared by extended straining, salt addition, and shaping into small balls, which are often preserved in olive oil. The process reduces moisture, concentrates fat and protein, and enhances storage stability while imparting characteristic lactic acidity [19,44,56].

Composition: Labneh Balls exhibit an average composition of 17.3% fat, 16.8% protein, and 60.3% moisture, with a pH of 4.1 and titratable acidity of 1.20% lactic acid [18,44,56].

Kg milk to kg Labneh: Producing 1 kg of Labneh Balls from cow milk typically requires 3.6 kg of 4% fat milk. For extra-dried goat Labneh with fat content exceeding 20%, milk requirements can reach 5.5 kg/kg product. GSO 816 mandates a minimum fat content of 12% for oil-preserved Labneh, implying at least 3.0 kg of milk is needed per kg of finished product [18,56].

Shelf Life: Labneh Balls preserved in oil exhibit significantly enhanced shelf life compared to fresh Labneh. The oil barrier, combined with low moisture and elevated acidity (~1.2% lactic acid), inhibits microbial growth and allows for storage at ambient temperatures for several months [56,57,60].

Classification: Despite their reduced moisture and firmer texture, Labneh Balls remain classified as “unripened fresh cheeses” in the Codex and Tetra Pak frameworks, owing to the absence of microbial or enzymatic ripening. However, their MFFB may fall below 67%, and their fat-in-dry-substance (FDS) content may approach or exceed 20%, suggesting a textural shift toward semi-soft or even semi-hard categories while retaining their fresh-cheese designation [53,61].

### 4.2. Labneh Anbaris

#### 4.2.1. Fresh Anbaris

Definition: Labneh Anbaris is a traditional Lebanese fermented dairy product prepared from raw cow or goat milk through a natural, uncontrolled fermentation process conducted in earthenware or glass vessels. This production method relies on successive additions of milk and salt over a period of 7–15 days at approximately 30 °C, with continuous draining of whey and refilling of the vessel until it is full. The resulting highly concentrated, acidic product is characterized by a dense texture, a strong tangy flavor, and extended microbiological stability without the addition of starter cultures or chemical preservatives. It is consumed either as a fresh, spoonable spread or shaped into small balls and preserved in olive oil to enhance flavor and storage stability [3,18].

Composition: Labneh Anbaris typically contains fat ranging from 15.0% to 16.17%, protein levels between 5.98% and 9.28%, and moisture content varying from 60% to 70%, depending on the extent of straining and fermentation. The pH values can drop as low as 3.5, reflecting its high acidity, which ranges from 1.62% to 2.0% lactic acid. The ash content has been reported between 2.8% and 3.7%, underscoring the product’s substantial mineral fraction derived from added salt and milk solids [3,18,19,62].

Kg milk to kg Labneh Anbaris: The production of one kilogram of Anbaris typically requires approximately 3.5 to 4.3 kg of 4% fat milk. This relatively high milk input is necessary to account for the extensive whey drainage and the prolonged, repeated fermentation stages inherent in the traditional artisanal process [3,18]. When shaped into small balls for oil preservation, the milk requirement generally ranges from 3.6 to 5.5 kg/kg of final dried product, depending on the milk type and desired fat concentration.

Shelf Life: Labneh Anbaris benefits from an extended shelf life compared to standard strained yogurts or fresh Labneh due to its low pH and high titratable acidity, which inhibit bacterial spoilage. When stored as fresh paste in sealed containers at refrigeration temperatures, it can last several weeks. In its traditional ball form, submerged in olive oil, it may be safely stored for many months at ambient temperature, with oil acting as an anaerobic barrier against spoilage organisms, though some risk of yeast or mold growth remains in high-moisture variants [3,18,19,58,60,62].

Classification: According to Codex Alimentarius CXS 243-2003 and the Tetra Pak Cheese Classification Matrix (2015) [53,59], Labneh Anbaris and its ball-formed variant are categorized as unripened fresh cheeses, given the absence of a dedicated curing stage. With fat-in-dry-substance (FDS) ranging from approximately 45% to just under 60% in the dried, ball-formed versions, they generally align with the full-fat category. The MFFB in plain Anbaris typically exceeds 67%, classifying it as soft cheese; however, the additional drying in the ball-formed variants may reduce moisture sufficiently to approach semi-soft characteristics. Despite these variations, both forms remain defined as uncured, fermented fresh cheeses under international standards.

#### 4.2.2. Labneh Anbaris Balls

Definition: Labneh Anbaris Balls are a traditional preserved form of Labneh Anbaris produced by further straining, salting, shaping, and storing the fermented paste in olive oil. This artisanal process concentrates solids, reduces moisture, and leverages the preservative properties of oil to extend shelf life without refrigeration. The technique reflects Lebanon’s longstanding adaptation to seasonal milk surpluses, ensuring a supply of dense, protein-rich dairy during periods of scarcity. These balls are typically handmade in rural households and represent a distinctive form of traditional dairy preservation [3,18].

Composition: Labneh Anbaris Balls generally contain fat ranging from 17.3% to 21.3%, protein content between 9.0% and 11.5%, and moisture levels reduced to approximately 45–60%, depending on the degree of straining and drying before oil preservation. Typical pH values are low, around 3.12, with a lactic acid content elevated to between 2.0% and 2.8%, contributing to the product’s distinctive sharp acidity and microbiological stability [3,18,62].

Kg milk to kg Labneh Anbaris Balls: Producing one kilogram of Labneh Anbaris Balls requires approximately 4.0 to 5.3 kg of 4% fat milk, depending on the milk type (cow or goat) and desired final fat concentration. The higher milk-to-product ratio results from intensive whey drainage and drying steps, which concentrate solids while ensuring a firm, cohesive texture suitable for shaping and long-term storage in oil [3,18,19].

Shelf Life: When preserved in olive oil, Labneh Anbaris Balls exhibit excellent shelf stability, often lasting for several months at ambient temperatures. The combination of reduced moisture, low pH, high lactic acidity, and the oil barrier effectively inhibits bacterial and fungal growth. This preservation approach traditionally allowed households to store dairy protein safely without refrigeration, aligning with the resource constraints of Lebanon’s mountain villages and semi-arid regions [3,18,19].

Classification: following the Tetra Pak Cheese Classification Matrix in Table 1, Labneh Anbaris Balls are categorized as unripened fresh cheeses despite their firm texture, due to the absence of any curing or enzymatic ripening phase. The intensive straining and drying process increases FDS to values typical of full-fat cheeses (≥45% and <60%), while the reduction in moisture may lower the MFFB sufficiently to approach semi-soft characteristics. Nevertheless, both Codex and GSO standards maintain them within the unripened fresh cheese category, recognizing their traditional production process as a variant of concentrated, fermented yogurt products [53,56,59]

### 4.3. Shanklish

#### 4.3.1. Fresh Shankleesh

Definition: Fresh Shankleesh (شنكليش طازج) is the early-stage, unripened form of this traditional Lebanese cheese. It is produced by heating yogurt to coagulate proteins, straining to remove whey, salting lightly, and shaping into balls for immediate or short-term consumption. Unlike the mature form, fresh Shankleesh does not undergo significant drying or mold ripening, resulting in a soft, spreadable texture with a mild, tangy flavor. It is typically served with tomatoes, onions, and olive oil as part of mezze platters [23].

Composition: Fresh Shankleesh generally contains approximately 65–68% moisture, 20–25% protein, and 3–5% fat, with a pH around 4.4–4.6 and titratable acidity between 1.0 and 1.2% lactic acid. These values reflect its high moisture and moderate acidity from yogurt fermentation with limited drying [4,23].

Kg milk to kg Product: Production typically requires 4.5–6 kg of milk per 1 kg of fresh Shankleesh, accounting for whey drainage during straining and partial moisture reduction [23].

Shelf Life: Due to its high moisture content, fresh Shankleesh is highly perishable and typically consumed within 7–10 days when refrigerated at 4 °C. The mild acidity provides limited microbial inhibition but does not substitute for cold storage [4,22,23].

Classification: According to the Tetra Pak Cheese Classification, as shown in Table 1, and Codex Alimentarius CXS 243-2003, fresh Shankleesh is classified as an unripened, acid-coagulated cheese with a high MFFB (>67%), placing it in the soft, low-fat cheese category [59].

#### 4.3.2. Mature Shankleesh

Definition: Mature Shankleesh (شنكليش معتق) is the fully ripened form produced by salting, shaping, sun- or air-drying, and aging yogurt-based curd balls for several weeks to months. The process reduces moisture, intensifies acidity, and promotes surface mold growth, which develops its strong, sharp aroma and crumbly texture. Traditionally coated with thyme, chili, or sumac, mature Shankleesh is served crumbled with vegetables and olive oil as a signature mezze dish [23,63].

Composition: Mature Shankleesh typically contains about 59–62% moisture, 30–33% protein, and around 2–3% fat, with a pH close to 4.1. Ripening lowers residual lactose while increasing water-soluble nitrogen and volatile acids, contributing to its sharp, savory profile [23,63].

Kg Milk to kg product: Producing 1 kg of mature Shankleesh generally requires 12–14 kg of milk. This high conversion ratio reflects extensive whey drainage and prolonged drying during aging, concentrating solids and reducing final moisture content [4,50].

Shelf Life: Properly ripened Shankleesh can be stored for several months to one year under cool conditions or in oil-sealed jars. The combined effects of low pH, salting, dehydration, and surface mold contribute to its preservation, while traditional spice coatings offer additional mild antimicrobial benefits [23,63].

Classification: According to the Tetra Pak Cheese Classification in Table 1, mature Shankleesh is classified as a ripened, acid-coagulated cheese. Its production involves yogurt acidification, heat treatment, salting, drying, and surface mold ripening, placing it in the low-fat, mold-ripened category within international classification systems [22,53,59].

### 4.4. Halloumi

#### 4.4.1. Fresh Halloumi

Definition: Fresh Halloumi (حلوم طازج) is a semi-hard, unripened cheese traditionally produced in Lebanon and the broader Eastern Mediterranean by coagulating milk, commonly sheep, goat, or mixed with cow milk, using rennet or acid-rennet methods. Production involves curd cutting, whey drainage, pressing, and scalding in hot whey without extended brine storage. The resulting cheese has a mild lactic flavor, moderate salinity from light surface salting, and a firm, elastic texture that resists melting, making it ideal for grilling or frying [33,34,64,65].

Composition: Fresh Halloumi typically contains approximately 43–48% moisture, 20–23% fat, and 22–26% protein, with a pH ranging from 5.9 to 6.4. The salt content is relatively low at 1–2%, reflecting minimal surface salting before sale [33,34,64,65].

Kg milk to kg product: Production generally requires about 11–12 kg of milk per kilogram of fresh Halloumi, depending on the milk type. Sheep and goat milk blends offer higher yield due to greater solids retention, while pure cow milk systems require more milk to compensate for a lower casein content [66].

Shelf Life: Fresh Halloumi is perishable and typically consumed within 10–14 days when stored under refrigeration at 4 °C. Without brining, salt levels are insufficient for long-term preservation, necessitating strict cold chain management to prevent spoilage [66,67].

Classification: According to the Tetra Pak Cheese Classification Table (2015) and Codex Alimentarius Standard CXS 283-1978 [53,59], fresh Halloumi is classified as an unripened, heat-treated, semi-hard cheese. Its moderate moisture-on-fat-free basis (~55–63%) places it within the semi-hard category, while scalding in whey differentiates it from purely acid-coagulated fresh cheeses.

#### 4.4.2. Brined (Mature) Halloumi

Definition: Brined or Mature Halloumi is prepared by pressing and cooking fresh curds and then immersing them in concentrated brine (typically 10–13% NaCl) for preservation. This brining process enhances microbial safety, intensifies saltiness, and firms the texture while maintaining Halloumi’s defining non-melting property, making it suitable for grilling, frying, or serving cold in salads and mezze [32,34,66].

Composition: Brined Halloumi typically contains 42–46% moisture, 22–25% fat, and 23–27% protein. The salt content increases substantially to 4–6% during brine storage, while the pH decreases slightly to approximately 5.5 [32,33,65,66,68,69].

Kg milk to kg product: The production of Halloumi cheese requires significant volumes of milk, with yield efficiency varying based on the milk type and coagulant used. According to El-Zoghby and Abdel-Kader (2000), approximately 8 kg of mixed buffalo and cow milk are needed to produce 1 kg of Halloumi [68]. Shchegolkov et al. (2022) reported a slightly higher requirement of 8.5 kg of cow’s milk per kilogram of cheese [65]. Meanwhile, Economides et al. (1987) demonstrated that milk yield efficiency differs based on the animal source, where 5.4, 8.85, and 11.5 kg of milk from sheep, goat, and cow, respectively, are needed per 1 kg of Halloumi [66].

Shelf Life: Brined Halloumi can be stored for several months to over one year at refrigeration temperatures. The combination of a high salt content, reduced pH, and controlled moisture ensures microbial stability and makes it suitable for distribution and storage in challenging climates [66,67,69,70].

Classification: According to the Tetra Pak Cheese Classification Table (2015) and Codex Alimentarius Standard CXS 283-1978 [53,59], brined Halloumi is an unripened, heat-treated, semi-hard cheese preserved in brine. Its heat treatment (scalding in whey), firm texture, and high salt content position it alongside other brined cheeses, like Feta, though it retains distinct regional methods and sensory profiles. This is in accordance with Kanafani (1981), who studied the processing of halloum and Lebanese melting cheese from reconstituted powder and fresh cow’s milk [32]. Its dual nature, both as a fresh and aged cheese, along with its heat resistance and brined preservation method, make it a standout product within Lebanese traditional dairy and a cheese of global culinary interest.

### 4.5. Karishi

Definition: Karishi is the Lebanese traditional equivalent of Mediterranean whey cheeses, particularly ricotta. It is produced by heating the residual whey from primary cheese-making, i.e., Halloumi and Akkawi byproducts, typically to 85–95 °C, inducing denaturation and coagulation of soluble whey proteins. This heat–acid coagulation method yields a fine-grained, soft, white curd that is skimmed from the whey and gently drained. The technique mirrors well-established practices in ricotta production across Italy and the broader Mediterranean region, with local adaptations that allow Karishi to valorize otherwise discarded whey streams and improve overall protein recovery [38,71,72,73].

Composition: Karishi’s composition is consistent with internationally documented specifications for whey-based ricotta cheeses. According to the USDA Dairy Division (1981) [73], standard whey-based ricotta contains approximately 72–74% moisture, 11–12% protein, and around 10% fat on a wet basis. Sakhala (2019) [74] specifically reported buffalo milk ricotta with 75% moisture, 11.67% protein, 0.145% titratable acidity (as lactic acid), and nearly 0.99 water activity, emphasizing its freshness and microbial vulnerability. Mangione et al. (2023) [38] reported a broader range for traditional Mediterranean ricotta varieties, with moisture contents typically between 65% and 75%, protein contents ranging from 8% to 12%, and fat contents near 10%. Empirical data from Bergamaschi et al. (2016) [72] confirmed these compositional parameters for artisanal whey-based ricotta produced in highland pasture systems, showing moisture contents of 72–76%, protein levels of 11–12%, and fat contents close to 10%. These consistent compositional values across studies underscore the alignment of Karishi with traditional whey-based ricotta profiles [38,71,72,74].

Kg milk to kg product: Karishi is made by recovering whey proteins from the whey left over after cheese-making. When rennet is used to coagulate milk into cheeses like Halloumi, about 11 L of milk produces 1 kg of cheese and around 9–10 L of whey. That whey, rather than being wasted, is reheated and acidified to yield Karishi, similar to Italian ricotta. According to data from ricotta production, about 4.5% to 5% of the whey volume is recoverable as cheese [72]. Thus, around 20 liters of whey are needed to produce 1 kg of Karishi. This variability reflects differences in whey solids concentration based on the primary cheese process, emphasizing Karishi’s role in valorizing nutrients that would otherwise be lost.

Shelf Life: Karishi is inherently a high-moisture, low-salt product, making it highly perishable. Without preservation treatments, its shelf life is typically 7–10 days under refrigeration [71,73]. Sakhala (2019) demonstrated that buffalo milk ricotta could be stored for up to 12 days in glass or polypropylene packaging, with its shelf life further extended to 21 days through the addition of essential oils and MicroGard™ [74]. These interventions underscore the need for careful packaging and storage strategies to maintain quality and safety in fresh whey cheeses [75].

Classification: Karishi’s lack of a ripening stage, its high moisture and low salt, and its production from residual whey proteins place it firmly within this category, reflecting a shared technological heritage with Mediterranean ricotta while retaining its distinct Lebanese identity. Karishi is best classified as an acid–heat-coagulated whey cheese, consistent with Codex-type categories for whey cheeses and USDA definitions of whey-based ricotta [73]. Both Mangione et al. (2023) [38] and Bergamaschi et al. (2016) [72] characterized ricotta as an unripened, fresh whey cheese produced through heat-induced denaturation and coagulation of soluble whey proteins. These definitions are consistent with the characterization of Karishi provided by the FAO (1990) [35]. Karishi thus shares key technological and compositional features with ricotta, exemplifying Lebanon’s adaptation of a broader Mediterranean cheese-making tradition while maintaining its unique cultural and culinary significance [38,72].

### 4.6. Pressed–Brine -Karishi (Lebanese Double-Cream)

Definition: Pressed–Brined Karishi is a Lebanese variation of traditional whey cheese, derived from Karishi through additional draining and pressing steps to reduce moisture and concentrate protein. Unlike standard fresh Karishi (analogous to Mediterranean ricotta), this product is prepared by pressing the freshly coagulated curd, sometimes with added cream and salt, to yield a firmer, sliceable texture with enhanced protein density. This technique aligns with well-documented Mediterranean practices for producing more compact, lower-moisture whey cheeses [38,71].

Composition: Pressed–Brined Karishi exhibits an elevated protein content compared to standard whey ricotta. While traditional ricotta and Karishi contain approximately 11–12% protein on a wet basis with 72–76% moisture [72,73], the pressed variant achieves approximately 17% protein with reduced moisture, estimated between 65% and 68% [76]. The same is performed for fat, which is around 15–17%. These values can be confirmed by the commercially sold Pressed–Brined Karishi (Lebanese Double-Cream), as stated on Carrefour Lebanon’s website [76].

Kg Whey to kg product: The yield efficiency for Pressed Ricotta Karishi is lower than for unpressed variants due to its higher solids content. Standard fresh whey ricotta production typically recovers about 4.5–5% of the whey volume as cheese, requiring around 20 kg of whey per kilogram of product [72]. For the pressed variant, with an ~48% higher protein concentration, the estimated whey requirement rises proportionally to approximately 40 kg per kilogram of finished product.

Shelf Life: Pressed–Brined Karishi benefits from reduced moisture and added salt, extending its shelf life relative to fresh Karishi. While traditional whey ricotta is typically consumed within 7–10 days under refrigeration [71,73], pressing and salting can allow storage for approximately 10–14 days under cold conditions unless vacuum packaged. Its high moisture still mandates continuous refrigeration and rapid consumption to prevent spoilage [38,70].

Classification: Pressed–Brined Karishi is classified as an acid–heat-coagulated whey cheese, consistent with Codex Alimentarius and USDA definitions for whey-based cheeses. It is produced via the heat-induced denaturation of whey proteins at near-boiling temperatures, followed by manual skimming, salting, and pressing. This process yields a fresh, unripened cheese with higher protein density and reduced moisture compared to standard Karishi, aligning it with global variants of enriched or pressed ricotta.

### 4.7. Qishta

Definition: Qishta (قشطة) is a traditional Lebanese dairy product produced by controlled heating of whole milk in wide, shallow vessels to form successive layers of coagulated milk skin. This artisanal process relies on gentle, prolonged heating—typically over two to three hours—to induce thermal denaturation and aggregation of milk proteins and fat at the liquid–air interface. As the milk simmers, thin, elastic films of coagulated proteins and fat repeatedly form on the surface. These layers are carefully lifted off, manually skimmed, and folded to create a semi-solid, creamy product with a distinctive layered structure [6,41].

Composition: Qishta typically contains approximately 11.7% fat, 12.1% protein, and 68% moisture, with a lactose content averaging 5.4% and an ash content around 1.6%. These values reflect a balanced distribution of denatured milk proteins and fat globules stabilized through heat-induced aggregation, resulting in a semi-dehydrated matrix with higher solids than the original milk [6]. The elevated protein content arises from the selective concentration of caseins and whey proteins at the heated surface, while the fat is coalesced and retained within the skimmed layers, creating the characteristic soft yet cohesive structure [40].

Kg Milk to kg Qishta: The production of one kilogram of Qishta typically requires between 5.5 and 7.5 kg of whole milk, depending on the heating protocol, the fat content of the starting milk, and the desired thickness of the collected skins. The relatively high conversion ratio reflects both the evaporation of water during prolonged heating and the selective removal of protein–fat layers from the milk surface, with the residual liquid often reused or fed to animals [6,41,42,43].

Shelf Life: Traditionally prepared Qishta is a fresh product with a limited shelf life, typically consumed within 3–5 days under refrigeration at 4 °C due to its high moisture content and minimal acidification. Microbial spoilage, especially from yeasts and psychrotrophic bacteria, limits storage even in refrigerated conditions. In artisanal settings, Qishta is generally produced daily or to order, ensuring freshness for confectionery use. Some modern adaptations involve mild pasteurization of the collected skins or modified atmosphere packaging to extend shelf life modestly without compromising texture [43].

Classification: According to Table 1 and Codex Alimentarius CXS 243-2003, Qishta is neither ripened nor unripened because it does not involve enzymatic coagulation, rennet, or lactic fermentation. Instead, Qishta is best categorized as a heat-concentrated milk product, akin to clotted cream or Kaymak. Its formation relies solely on thermal denaturation and aggregation of caseins and whey proteins at the surface of heated milk, with no added cultures or ripening phase. While its moisture content (~68%) and fat content (~11.7%) would numerically place it near some soft or high-moisture cheese categories if forced into the Tetra Pak matrix, its production method and sensory properties put it in a special category of its own. Tetra Pak and Codex standards thus consider such products outside the formal cheese classification, recognizing them instead as traditional concentrated dairy preparations that occupy a parallel but distinct space in the dairy taxonomy [6,53,59].

### 4.8. Kishk

#### 4.8.1. Fresh Kishk

Definition: Fresh Kishk is a traditional Lebanese fermented food produced by blending strained yogurt with bulgur wheat, allowing lactic fermentation to stabilize the mixture and develop characteristic acidity and flavor. It is typically consumed as a moist paste shortly after production [16,44,45,77].

Composition: The standard fresh Kishk formulation uses approximately 3.2 kg of yogurt and 0.8 kg of bulgur to yield about 4 kg of product. Its typical composition includes 8–10% protein, 4–6% fat, and up to 25% moisture, with acidity levels between 1.3% and 1.4% lactic acid. The milk source influences final composition: goat milk is similar to cow milk in solids, while sheep milk offers ~1.5 times higher solids [16,44,45,77].

Kg milk to kg Product: Approximately 3.2 kg of yogurt plus 0.8 kg of bulgur yields 4 kg of fresh Kishk. When using milk to produce Kishk, 0.8 kg of milk yields 1 kg of Kishk. Variations arise from the yogurt source: if Greek yogurt/labneh is used, the source is cow, sheep, or goat [16,25,44,45,77].

Shelf Life: Fresh Kishk benefits from its acidity for microbial stability but remains perishable. When preserved in oil, the water activity can drop to ~0.62, extending its shelf life without refrigeration [44,77].

Classification: Following the criteria in Table 1, fresh Kishk is a fermented composite dairy–cereal food, functioning as a high-moisture, probiotic-rich product suitable for short-term storage and local consumption.

#### 4.8.2. Dried Kishk

Definition: Dried Kishk is produced by dehydrating the fermented yogurt–bulgur mixture to create a shelf-stable powder with highly concentrated nutrients and extended storage capability [16,44,45,78,79,80,81,82].

Composition: The moisture content is reduced to 2.9–3.5%, the acidity rises to ≥4.5% lactic acid, and the pH drops to ≤3.95. Dried Kishk typically contains 23–25% protein, 13–14% fat, 3.5–4.0% ash, and water activity between 0.34 and 0.4 [16,44,45,78,79,80,81,82,83].

Kg milk to kg product: Using the same initial mixture (3.2 kg yogurt + 0.8 kg bulgur), the dehydration process yields ~1 kg of dried Kishk per 1 kg of fermented paste [16,45,78,79,84].

Shelf Life: Its low moisture, high acidity, and reduced water activity enable ambient storage exceeding one year, supporting both household use and export markets [45].

Classification: Following Table 1, dried Kishk is a shelf-stable, fermented dairy–cereal powder, aligning with traditional functional foods valued for nutrient density, microbial safety, and long-term storage without refrigeration [45].

## 5. Discussion and Synthesis

### 5.1. Interpretation of Nutritional Composition and Conversion Efficiency

Traditional Lebanese dairy products exhibit considerable variation in nutritional composition and production efficiency, reflecting both cultural preferences and adaptations to local resource constraints. A clear understanding of these differences is essential for evaluating their potential role within modern food systems, regulatory frameworks, and sustainability strategies.

Table 2 presents the nutritional composition of the principal traditional dairy products analyzed in this study. Key parameters, including fat content, protein concentration, moisture levels, salt content, pH, and titratable acidity, highlight the diversity of products ranging from fresh to preserved and fully dehydrated varieties. These compositional characteristics directly inform classification under the Codex Alimentarius and Tetra Pak systems (as outlined in Table 1) and provide important indicators of each product’s suitability for different market segments.

The data presented in Table 2 illustrate a compositional continuum ranging from high-moisture and low-salt products, such as fresh Labneh and Karishi, to reduced-moisture, higher-salt, and oil-preserved varieties, including Labneh Balls, Shanklish, and dried Kishk. This variability supports the diversification of traditional dairy production pathways, enabling both short-term local consumption and longer-term storage in the absence of refrigeration. Such adaptability represents an important resilience mechanism in contexts where cold chain infrastructure is limited or unreliable. In addition, the elevated protein and fat concentrations observed in the preserved products contribute to increased nutritional density, positioning these items as nutrient-dense products with high market value. This enhances their potential appeal for both domestic markets and targeted export opportunities.

Table 3 consolidates conversion ratios from milk to product (or from whey to product), alongside classification categories. These yield efficiencies are essential for assessing the sustainability and economic viability of traditional dairy processing, particularly under conditions of resource constraint.

As demonstrated in Table 3, the conversion ratios for traditional Lebanese dairy products are generally comparable to those observed in industrial dairy systems, particularly when the valorization of byproducts, such as whey, is taken into account. The transformation of whey into Karishi and its pressed, salted form, referred to in local terminology as Double-Cream, illustrates a resource-efficient production model that aligns with the principles of a circular economy. In a similar manner, the incorporation of cereal in Kishk production enhances both caloric density and nutritional value while simultaneously reducing reliance on imported inputs.

The data presented support the argument that traditional Lebanese dairy products are not merely artisanal or heritage-based but technically robust and adaptable elements within sustainable food systems. They provide a practical foundation for regulatory standardization, quality certification, and branding strategies that could facilitate modernization without compromising cultural authenticity. The integration of these products into national food security planning, supported by targeted assistance to small-scale producers, has the potential to enhance economic resilience while preserving Lebanon’s rich culinary and gastronomic heritage.

### 5.2. Comparative Analysis of Traditional Lebanese Cheeses

The classification and conversion efficiency of traditional Lebanese cheeses reveal a distinctive profile when analyzed both internally and against international benchmarks. Lebanese cheeses predominantly fall within the unripened or semiripened categories, typically characterized by a high moisture content, artisanal production techniques, and notable compositional variability. Cheeses such as Labneh, including its oil-preserved ball form (Makbouseh), Pressed–Brined Karishi (known as Double-Cream in Lebanon), and Karishi demonstrate relatively efficient milk-to-cheese conversion ratios, requiring approximately 2.5 to 3 kg of milk per kilogram of final product. These values align with international standards for soft, fresh cheeses like Quark and ricotta and conform to the Codex Alimentarius and Tetra Pak classification frameworks for unripened dairy products in terms of moisture and fat content [53,61].

Conversely, cheeses such as Halloumi, Shanklish, Anbaris (including its preserved ball form), and particularly Kishk exhibit lower conversion efficiencies, often requiring 5 to 14 kg of milk per kilogram of product.

While such inputs are consistent with hard or aged cheeses in international systems (e.g., Manchego or Parmesan [89,90]), Lebanese varieties are usually less extensively ripened. They are produced primarily for local or seasonal consumption rather than long aging or large-scale export.

Anbaris occupies a unique position within this landscape as a fermented yogurt concentrate traditionally made from strained yogurt, often sheep or goat milk, and aged in clay jars for extended periods. Over time, natural fermentation and dehydration yield a thick, salted, and concentrated dairy paste used historically as a preservation method in rural and highland communities. Although its exact yield can vary, Anbaris typically requires a high milk input per unit of product, placing it on the lower-efficiency end of the conversion spectrum. However, its prolonged shelf life, cultural role in food preservation, and intense flavor profile distinguish it from typical ripened cheeses, positioning it between a functional ingredient and a fermented dairy reserve.

Kishk represents an especially distinctive case that diverges from classical cheese definitions. It is a fermented dairy–cereal blend made by mixing yogurt with bulgur wheat, allowing fermentation, and subsequently sun-drying and grinding the mixture into a low-moisture powder with approximately 4 percent moisture content [16,45,79,91]. While the dairy input is moderate, approximately 5 kg of milk per kilogram of final product, its hybrid matrix, powdered form, and minimal water content, <10% moisture, preclude it from standard cheese categories. Instead, Kishk is best classified as a dried, fermented dairy–cereal product that illustrates the limitations of rigid global cheese taxonomies when applied to regionally distinct, multifunctional foods.

In terms of compositional attributes, products such as Labneh, Halloumi, Pressed–Brined Karishi (Lebanese Double-Cream), and Karishi most closely meet international standards for moisture, fat, and salt levels. For example, Lebanese Halloumi, while analogous to Cypriot Halloumi in its brining and grilling function, undergoes a less extensive stretching phase, resulting in a slightly softer texture and higher moisture content, which affects both its shelf life and functional behavior [64]. Other cheeses, including Shanklish and Anbaris with its ball form, exhibit substantial variability in compositional parameters, largely due to traditional fermentation environments, artisanal salting, and drying conditions.

Although not a cheese in the strict sense, Qishta, produced through repeated heating and skimming of milk to form layered milk skin, shares artisanal, high-moisture, fresh-dairy characteristics that complicate simple classification. Its inclusion in this analysis underscores the broader spectrum of Lebanese dairy tradition, from spreadable and pressed cheeses to specialized, regionally distinctive products.

This compositional and classificatory heterogeneity reflects both a challenge and an opportunity. While it complicates alignment with international certification systems that require standardization and reproducibility, it also signals a rich diversity of artisanal practices and terroir-linked knowledge systems. With targeted compositional characterization and selective standardization, these cheeses, especially lesser-known types such as Kishk, Anbaris, and Shanklish, have the potential to be positioned within niche heritage markets, offering both gastronomic value and cultural continuity.

### 5.3. Traditional Versus Industrial Production Practices

The contrast between traditional and industrial production systems is central to understanding the current structure and future potential of Lebanese dairy products. Traditional Lebanese cheese-making is deeply rooted in household and rural practices that emphasize spontaneous fermentation, manual processing, and low-intervention preservation methods. These approaches produce rich sensory diversity and cultural authenticity but also present systemic inefficiencies such as relatively low conversion yields for products like Shanklish, Kishk, and Anbaris Balls, where extended drying or fermentation leads to substantial mass loss; labor-intensive methods requiring skilled manual operations that limit scalability; and high product variability arising from unstandardized microbial ecosystems, fluctuating milk quality, and informal production environments.

Products such as Kishk, Anbaris, and Shanklish exemplify preservation strategies specifically adapted to Lebanon’s climate and food culture. Kishk is a fermented dairy–cereal blend that combines yogurt with bulgur wheat and then undergoes sun-drying to produce a stable powder with less than 7 percent moisture. Although its milk input is moderate at roughly 4–5 kg per kilogram of final product, the addition of bulgur, extensive drying, and its powdered form place it outside conventional cheese categories. It remains a staple of family-based production, valued in rural areas for its storability, nutrient density, and use as a culinary base throughout the year.

Anbaris, by contrast, is a fermented dairy concentrate produced solely from salted milk that is strained and aged in clay jars for several weeks. This method is particularly associated with Lebanon’s mountainous regions, where refrigeration has historically been scarce, necessitating the transformation of fresh milk into dense, acidic, high-salt forms suitable for long-term storage [3,18,19,62]. Both Anbaris and its oil-preserved ball variant embody these preservation strategies while maintaining strong ties to geographic identity and local ecological knowledge.

Other cheeses such as Labneh, Labneh Balls, Halloumi, Karishi, Pressed–Brined Karishi (known locally as Double-Cream), and Qishta also demonstrate artisanal traits, though with varying degrees of transformation and preservation. Fresh Labneh involves yogurt-straining and is consumed immediately or preserved in oil as Labneh Balls to extend its shelf life. Qishta is produced by carefully skimming coagulated milk skin from gently heated whole milk, creating a fresh, high-moisture dairy layer with minimal intervention. Karishi and Pressed–Brined Karishi (Lebanese Double-Cream) exemplify whey valorization, converting residual liquid into spreadable or pressed forms through gentle heating, pressing, and salting to enhance shelf stability. Shanklish undergoes ball formation, surface fermentation, and drying or mold-ripening, often coated with herbs to develop its distinctive firm texture and robust flavor. Halloumi is a semi-hard, brined cheese produced through stretching and folding techniques that make it suitable for grilling while extending its shelf life through brining. Akkawi, a similar white brined cheese, is traditionally made without stretching, and its mature brined form is sometimes referred to locally as “Akkawi Tscheeky” because of imports from Czech producers who supplied the regional market at competitive prices.

By contrast, industrial production methods introduce consistency, safety, and efficiency. Techniques such as pasteurization, ultrafiltration, standardized starter cultures, and controlled salting or pressing enable higher yields through improved moisture retention and whey recovery, ensure better hygiene and shelf life via controlled microbial environments, and allow for commercial scalability and wider distribution both locally and for export.

Several traditional cheeses, most notably Labneh, Halloumi, Karishi, and Pressed–Brined Karishi (Lebanese Double-Cream), have already been successfully integrated into hybrid production systems that combine artisanal sensory attributes with industrial precision. In these cases, essential qualities, such as tanginess, texture, salinity, and preservation, are maintained while processing conditions are adapted to meet food safety standards and market demands.

Promoting such hybrid models could benefit a wider range of products, including less industrialized types, such as Shanklish, Anbaris, and Qishta, through targeted investments in rural infrastructure, micro-dairy facilities, and technical training. Supporting standardized yet culturally sensitive production models would help ensure economic sustainability while safeguarding the culinary heritage embodied in Lebanon’s diverse dairy repertoire. For comparisons of yield, labor, and production times across methods, see Table 4.

### 5.4. Cultural–Sensory Dimensions of Cheese Classification

Cheese classification systems serve not only technical and regulatory functions but also reflect embedded cultural and sensory values. In the Lebanese context, traditional dairy products are deeply tied to culinary customs, regional geography, and seasonal production cycles, embodying centuries of local adaptation and the transmission of knowledge. Many of these cheeses have been formally identified in an official inventory issued by the Lebanese Ministry of Economy and Trade. Supported by EFTA and Swiss development cooperation, this documentation highlights their unique production methods, regional specificity, and cultural heritage with the aim of securing PGI status [24].

Labneh and its preserved form, commonly known as Makbouseh, are among the most widely consumed traditional dairy products in Lebanon. Their creamy and tangy profile is central to daily meals, especially during breakfast and within mezze platters. Regional variants, such as Labneh Taenayel, Labneh Chtoura, and Labneh Darf Baalbeck, reflect differences in milk origin, fermentation time, and preservation technique. Labneh Anbaris, referred to in some areas as Sirdalé, is traditionally fermented and stored in earthenware jar containers. The result is a dense, acidic product with extended shelf stability, which is well suited for winter consumption.

Karishi, which can be described as Lebanese ricotta, and its pressed and salted variant, locally known as Lebanese Double-Cream, illustrate the transformation of whey into soft, spreadable dairy products. These cheeses embody resource-conscious dairy practices that valorize byproducts while offering accessible and protein-rich foods suitable for both household and small-scale production.

Qishta represents a distinct category of fresh dairy products, prepared by skimming successive layers of coagulated milk skin during gentle heating. Its delicate texture and mild sweetness contribute to its use in both breakfast dishes and desserts. The product reflects artisanal skill and minimal technological intervention.

Anbaris, including its oil-preserved form, serves as an example of fermented and salted fresh cheese intended for long-term storage. Traditionally aged in earthenware containers, Anbaris develops a dense structure, strong acidity, and notable salinity. These properties facilitate preservation in mountain communities with limited access to refrigeration and reflect the influence of local microflora and salting traditions.

Halloumi is a semi-hard, brined cheese recognized for its elastic and squeaky texture, which allows it to retain its structure during grilling and frying. Lebanese Halloumi shares many attributes with the Cypriot version, but it often contains a higher moisture content and a softer texture due to differences in milk composition and regional processing preferences. Akkawi, another important white brined cheese, is traditionally produced without stretching. It is typically consumed fresh or in firmer, aged forms. The mature variant is sometimes referred to as Akkawi Tscheeky, a term that originated following the import of similar cheeses from Czech producers and now serves as a colloquial market identifier.

A related preservation practice known as Darfiyeh involves packing soft white cheeses, such as Akkawi or Halloumi, into a sealed natural container (darf), where they are aged slowly. This method concentrates salt, firms texture, and extends shelf life in the absence of refrigeration. It demonstrates an artisanal approach to natural fermentation that has been formally recognized in Lebanese PGI initiatives [24].

Shanklish, especially that produced in Rahbeh in North Lebanon, is a mold-ripened cheese formed into balls and coated with herbs, such as thyme or chili. It undergoes extended fermentation and drying, producing a granular texture and a robust, evolving flavor profile. As a central element in the mezze tradition, Shanklish exemplifies regional culinary identity and long-standing artisanal expertise.

Kishk occupies a unique position within traditional food systems. It is a dried fermented blend of yogurt and butghul that does not fit conventional cheese categories. Characterized by pronounced acidity, high nutritional value, and extended shelf stability, Kishk has historically served as a winter staple in rural diets. It functions as both a preserved nutrient source and a flavoring agent in soups and stews, representing a food security adaptation shaped by Lebanon’s climatic and economic conditions.

The diversity of forms, textures, and fermentation profiles represented by these products highlights the need for classification frameworks that integrate technological attributes, such as ripening status, fermentation method, and moisture content, with cultural meaning and sensory characteristics. Such integrative systems facilitate regulatory alignment while preserving Lebanon’s intangible culinary heritage. These cheeses are not merely food commodities but represent expressions of local knowledge, ecological adaptation, and culturally rooted taste. They warrant formal protection, institutional support, and investment in product differentiation and development.

Although several of Lebanon’s traditional dairy products have been documented for their heritage and regional distinctiveness, most efforts remain in a preparatory or advocacy phase. Legal frameworks for PGI registration are not yet fully operational or enforced. Existing initiatives primarily serve as foundational inventories rather than as tools for enforceable market protection. Bridging this gap will require coordinated policy reform, institutional support, and capacity building among producer associations.

These efforts may be further reinforced through alignment with Lebanon’s Draft Environmental Guidelines for the Dairy Industry [92], which remain under development. These guidelines already establish standards for site zoning, pollution control, and hygienic facility design. Integrating Protected Geographical Indication systems within such environmental governance frameworks can strengthen their institutional legitimacy and create a multisectoral compliance pathway that links cultural preservation with enforceable regulatory structures.

### 5.5. Systemic Challenges and Contextual Resilience

While traditional Lebanese dairy products offer cultural depth, nutritional value, and artisanal identity, they are produced within a context of significant structural challenges. Lebanon currently imports an estimated 70–80% of its dairy supply [2], driven by high domestic production costs, reliance on imported feed, and underdeveloped cold chain infrastructure [10]. Political and economic instability further disrupts dairy farming, impeding local milk production, investment, and distribution logistics.

In this challenging environment, traditional cheeses and preserved dairy products gain renewed importance as resilient, low-tech solutions. Their value lies not only in cultural continuity but also in reduced dependence on refrigeration, extended shelf lives, and suitability for small-scale, decentralized production models. For instance, Kishk, as a fermented dairy–cereal powder with a moisture content as low as 4%, provides a stable, non-refrigerated reserve that lasts more than a year, mitigating the need for cold storage or daily milk collection and serving as an essential winter staple in rural diets.

Shanklish, Anbaris Balls, and Matured Labneh Balls further exemplify these preservation strategies. Through salting, drying, oil preservation, or mold-ripening, these products maintain quality and safety over extended periods, even under suboptimal storage conditions. Many of these preservation strategies function by reducing water activity to levels below 0.85, inhibiting bacterial growth, or even below 0.7, to suppress fungal and mold spoilage entirely, while maintaining acidity levels below pH 4.6 to qualify as high-acid foods with enhanced microbial safety. Anbaris itself, with its ball form preserved in oil or stored in brine in clay jars, demonstrates how traditional fermentation and salting can extend usability without advanced technology.

Other products, such as Karishi and Pressed–Brined Karishi (Lebanese Double-Cream), highlight circular economy practices by transforming whey, a byproduct, into high-value cheeses through simple heating, skimming, and pressing processes, with added salt for preservation. These methods reduce waste, improve protein recovery, and require minimal technological infrastructure, aligning with sustainability goals even in low-resource contexts.

Even fresh products like Labneh, Labneh Balls, Halloumi, and Qishta, though more reliant on short-term cold storage, offer important flexibility. Labneh can be consumed fresh or preserved as balls in oil for months, while Halloumi’s brining extends its shelf life and allows for safe transport. Qishta, although highly perishable due to its very high moisture content, can be produced quickly from fresh milk with minimal equipment, making it accessible to households with limited infrastructure.

Critically, the effectiveness of these traditional preservation strategies can be understood through their impact on water activity (aw). As Marcos et al. (1981) demonstrated, in cheeses with a high moisture content, aw can be reliably predicted from salt concentration, while in lower-moisture varieties, the accumulation of solids and proteolysis further depresses aw [93]. This explains why products like dried Kishk achieve aw levels below 0.7, effectively suppressing fungal and mold spoilage, while oil-preserved Labneh Balls or Anbaris Balls maintain aw below 0.85, inhibiting bacterial growth even without refrigeration. Combined with maintaining pH below 4.6—a defining feature of high-acid foods—these properties ensure microbial safety and extended shelf life [94].

This adaptability underscores the strategic role of Lebanon’s traditional dairy heritage in building resilience during periods of economic uncertainty or infrastructural weakness. Other products, such as fresh Akkawi and its mature (Akkawi Tscheeky) form, illustrate similar brined preservation strategies to Halloumi, relying on salt and controlled storage to extend shelf life without advanced technology. By modernizing hygiene standards and optimizing yield without compromising artisanal methods, these systems can not only preserve cultural heritage but also bolster national food security. Supporting small-scale producers through training, investment in rural infrastructure, and policy incentives can help maintain this rich dairy tradition while reducing reliance on imports and enhancing local economic sustainability.

Storage durations and preservation methods for these diverse products are summarized in Table 5, highlighting varied approaches, ranging from refrigeration to drying, oil preservation to brining, and fermentation, enabling these cheeses to remain viable under different environmental and economic conditions.

### 5.6. Economic and Sustainability Modeling

The economic performance and ecological footprint of traditional Lebanese dairy products are closely tied to milk-to-product conversion efficiency, whey management practices, and the types of milk employed. A detailed examination of yield dynamics, byproduct utilization, and strategic resource use reveals significant opportunities to align artisanal production with sustainability objectives without sacrificing cultural integrity.

#### 5.6.1. Yield Efficiency and Cost Implications

Cheese yield plays a crucial role in determining production costs and market viability, particularly in regions with constrained milk availability or seasonal variation in supply. Products such as fresh Labneh, Lebanese Double-Cream, Karishi, Akkawi, and Qishta demonstrate relatively high conversion efficiencies, typically requiring only 2.5 to 3 kg of milk per kilogram of final product. These yields match international benchmarks for soft, fresh cheeses and support low-cost, high-turnover business models appropriate for both domestic and export markets.

In contrast, products such as Shanklish, Dried Kishk, Anbaris, and their respective ball forms present lower efficiency, with milk inputs ranging from 5 to 14 kg per kilogram of final product. These lower yields result from extended fermentation, pressing, drying, or preservation stages that are essential to their artisanal character. Shanklish, for example, requires careful ball formation, salting, surface fermentation, and often herbal coating, processes that increase input costs and labor intensity but yield a distinctive, culturally valued product. Dried Kishk combines yogurt and bulgur and then undergoes significant dehydration, reducing net yield while increasing shelf life and nutritional density. Labneh Anbaris and its oil-preserved balls also fall within this lower-yield category due to their prolonged fermentation and straining in earthenware jars, practices that demand premium pricing or protected designation to remain economically viable.

Halloumi occupies an intermediate position in this economic landscape. Fresh Halloumi offers relatively favorable yield and rapid marketability, while matured and brined Halloumi involves longer production time, careful salting, and storage, increasing handling costs but delivering extended shelf life and export suitability.

The key insight is that high-yield products, like fresh Labneh and Pressed–Brined Karishi, or Lebanese Double-Cream, are economically critical in low-resource contexts, whereas low-yield items, such as Shanklish, Dried Kishk, and Anbaris-based cheeses require, niche marketing strategies and added-value labeling to justify artisanal production costs. While some short-term strategies may rely on imported milk powders or liquid milk to offset domestic shortages, such dependence, already evident in trade patterns documented by FAOSTAT (2023) [1], introduces vulnerability to price volatility and supply shocks. Conversely, enhancing local ruminant-based systems offers a more resilient pathway aligned with Lebanon’s agro-ecological potential and cheese-making traditions.

#### 5.6.2. Whey Valorization and Circular Economy Practices

Another critical element of sustainable dairy economics in Lebanon is whey valorization. Whey typically accounts for 40 to 50 percent of the milk volume after curd extraction and represents both an environmental challenge and a strategic resource. Several Lebanese products exemplify effective circular resource utilization by transforming whey into high-value cheeses such as Karishi and Lebanese Double-Cream. These methods are not only traditional but are also consistent with modern zero-waste dairy production models adopted globally [95], improving overall resource efficiency and contributing to nutritional diversity. Residual whey can also be reintegrated into fermentation bases for Fresh Kishk, enhancing its protein content and microbial complexity, as suggested by Dimassi, 2025 [16], further reinforcing low-waste principles.

Investments in whey collection systems, hygienic handling, and dehydration technologies could help scale these valorization practices, particularly for small-scale rural producers seeking to convert perishable byproducts into shelf-stable, marketable goods. Such approaches align with global sustainability targets, including SDG 12.3, which calls for significant reductions in food loss and waste throughout production and supply chains [96].

#### 5.6.3. Strategic Utilization of Sheep Milk

Strategic utilization of sheep milk also offers important sustainability and economic advantages. Sheep milk contains higher total solids, notably protein and fat, resulting in yields up to 1.8 to 2 times higher than cow milk on a per-liter basis. This characteristic is especially beneficial for products that require extensive fermentation or drying, such as Dried Kishk, Shanklish, and Anbaris Balls, where solid retention directly affects yield and quality.

Promoting sheep milk production in Lebanon’s mountainous and semi-arid regions not only enhances efficiency but also supports rural resilience, given that small ruminants are better adapted to local topographies and forage conditions than cattle. Encouraging this strategy could alleviate pressure on bovine milk supplies in coastal and peri-urban areas, support agropastoral livelihoods, preserve traditional grazing practices, and contribute to biodiversity and soil conservation.

#### 5.6.4. Strategic Utilization of Goat Milk

Goat milk production in Lebanon, particularly from the Baladi breed (which accounts for over 96% of the country’s goat population), offers critical sustainability and economic advantages in traditional dairy production. Baladi goats are well adapted to Lebanon’s mountainous and semi-arid regions, tolerating poor forage quality and seasonal variability [97]. Their milk is distinguished by relatively high total solids, including ~4.3% fat and ~4.0% protein, which makes it highly suitable for cheese-making with good yield potential comparable to or exceeding that of cow milk under many conditions.

Experimental cheese-making trials in Lebanon have demonstrated that Baladi goat milk can produce ripened cheeses with good hygienic and sensory quality using selected starter cultures, even in small-scale farm settings [97]. Such capacity expands opportunities for rural producers to diversify beyond short-shelf-life fresh products, supporting income stability and reducing dependence on imported dairy.

Encouraging the use of goat milk in artisanal and semi-industrial production aligns with sustainability goals by supporting agropastoral livelihoods, preserving traditional grazing practices that enhance biodiversity, and promoting local value addition. Moreover, the development of branded, protected, or geographically indicated goat milk cheeses can open niche markets both domestically and abroad, enhancing Lebanon’s food sovereignty and economic resilience.

#### 5.6.5. Shelf Life as an Economic and Sustainability Lever

While high-yield products, such as fresh Labneh, Fresh Halloumi, Akkawi, Karishi, Pressed–Brined Karishi—Lebanese Double-Cream, and Qishta are economically critical in low-resource contexts due to their short production times and relatively low milk inputs, lower-yield items, like Shanklish, Dried Kishk, and Anbaris, require differentiated marketing approaches. These artisanal products trade yield for longevity, complexity, and cultural capital. They demand premium pricing, niche marketing, and clear heritage branding to justify their input costs.

Shelf life is a particularly important economic and sustainability lever. While products such as fresh Labneh, fresh Halloumi, fresh Akkawi, and Qishta require prompt consumption or refrigeration, aged or preserved varieties, including Shanklish, Dried Kishk, Matured Labneh Balls, Anbaris Balls, and brined Halloumi, offer extended shelf lives ranging from several months to over a year. This extended shelf stability offers multiple advantages:

- Reduced Post-Harvest Losses: A long shelf life lowers the risk of spoilage, particularly in rural or under-resourced areas lacking cold chains.
- Market Flexibility: Producers can sell these goods over time, responding to price fluctuations or market demand rather than rushing to market immediately after production.
- Seasonal Smoothing: Products like Dried Kishk and Anbaris Balls are traditionally made during surplus seasons (spring and early summer) and consumed in winter, effectively functioning as nutritional reserves and stabilizing rural food security.
- Export Potential: Shelf-stable formats are better suited for regional and international trade, making them strong candidates for diaspora markets and heritage branding.

Therefore, the true economic value of traditional Lebanese dairy products cannot be evaluated solely through immediate yield per kilogram of milk but must also account for shelf life, storability, processing time, cultural value, and market flexibility. Integrated economic modeling should consider these multiple dimensions, recognizing that lower-yield, higher-labor cheeses often deliver greater resilience and sustainability over time by stabilizing rural incomes, reducing waste, and preserving Lebanon’s culinary heritage.

Furthermore, supply chain governance factors, such as contract duration, on-farm inspections, and the choice of trading partners, were shown to significantly influence pathogen risk and the likelihood of adopting Good Agricultural Practices (GAP)-compliant practices [52]. Despite the dominance of informal agreements in Lebanon’s dairy sector, these findings suggest that promoting formalized contracts and public–private oversight mechanisms could simultaneously enhance food safety and economic resilience.

### 5.7. Future Directions and Research Gaps

Despite the rich diversity and deep cultural significance of Lebanon’s traditional dairy products, important research gaps remain that warrant systematic investigation. Detailed compositional analyses using modern analytical methods could help standardize nutrient profiles and support clearer classification frameworks that align with international regulations while preserving local specificity. There is also a need for longitudinal studies on microbial ecology in traditional fermentation processes, especially for products like Anbaris, Shanklish, and Kishk, to ensure food safety while maintaining artisanal qualities.

Economic studies examining the viability of scaling up traditional production using hybrid artisanal–industrial models are limited. Research on rural supply chains, input costs, and market opportunities, including export and diaspora markets, could help design policies that support small-scale producers without compromising traditional methods. The role of sheep and goat milk in enhancing yield efficiency and sustainability also deserves more quantitative modeling, particularly in the context of climate adaptation and agropastoral livelihoods. Although the present review focuses on the most commonly documented traditional dairy products, the literature remains incomplete. Products such as Darfiyeh, Baladi cheese, and regional Labneh variants remain under-studied in compositional, microbiological, and economic terms. These gaps highlight the need for field-based documentation and laboratory validation to support future regulatory inclusion and heritage protection.

One particularly underdeveloped area is the operationalization of innovation and policy frameworks in support of traditional dairy systems. Among the 96 sources reviewed for this study, only five directly addressed themes such as innovation policy, cooperative development, or GI protection within Lebanon’s dairy sector—including Nehme et al. (2019) [23], Jalkh et al. (2020) [11], Tarapoulouzi et al. (2024) [34], Al Kadi (2021) [48], and Dal et al. (2021) [10]. While additional technical studies, such as Abebe et al. (2017) [52], provide insights into food safety governance and FSMS implementation, they do not extend to the valorization of traditional products or PGI mechanisms. Similarly, the LMOE (2016) [92] Draft Environmental Guidelines for the Dairy Industry focus on regulatory infrastructure but do not address cultural–geographic protections. However, these contributions remain largely conceptual, offering descriptive insights without detailing implementation strategies, financing models, or institutional pathways. While the MOET (2007) [24] inventory provides a valuable foundation for PGI documentation in Lebanon, it constitutes an initiative rather than an academic study. This highlights a significant research gap and an opportunity for future interdisciplinary work that integrates cultural preservation with innovation-driven food system transformation.

Additionally, further work on hygienic processing innovations, low-cost preservation technologies, and training systems for rural producers could enhance quality assurance while maintaining accessibility. A key opportunity lies in transitioning from short-term inspections to long-term governance systems that incentivize good practices. Public–private partnerships can support the adoption of farm-level FSMSs such as GAP, particularly where gaps in milking hygiene and water handling remain severe. As shown by Abebe et al. (2017) [52], even basic contractual formalization, when paired with targeted training and credit support, can help overcome adoption barriers among smallholders. Embedding these mechanisms in regional dairy strategies would bridge the current divide between food safety law and rural implementation.

Finally, consumer studies exploring sensory preferences and willingness to pay for traditional versus standardized variants could guide branding, marketing, and protected designation efforts, helping these unique products secure their place in both local diets and international markets. Given the strong overlap in dairy heritage across the Levant and Eastern Mediterranean, including Syria, Jordan, Cyprus, and other countries in the region, future studies and policy frameworks could explore opportunities for cross-border collaboration in traditional dairy classification, innovation platforms, and mutual recognition of quality schemes (e.g., PGI/PDO), enabling Lebanon’s dairy sector to benefit from shared regional expertise and diaspora market synergies.

## 6. Conclusions

This review has provided a detailed compositional, classificatory, and economic analysis of Lebanon’s traditional dairy products, encompassing fresh Labneh, Labneh Balls (Makbouseh), Labneh Anbaris, Anbaris Balls, Shanklish in both its fresh and mature forms, Halloumi in fresh and brined versions, Karishi (Lebanese Ricotta), Pressed–Brined Karishi (Lebanese Double-Cream), Qishta, and both fresh and dried forms of Kishk. Products such as fresh Labneh, Karishi, Pressed–Brined Karishi (Lebanese Double-Cream), and Qishta achieve relatively high yield efficiencies and align well with international standards for moisture, fat, and salt content, making them suitable for integration into modern food systems with minimal adaptation.

In contrast, items like Shanklish, Dried Kishk, Anbaris, and their oil- or brine-preserved variants offer extended shelf lives and rich cultural specificity at the expense of immediate yield efficiency. These trade-offs are not merely technical choices but reflect a broader strategy of resilience and adaptation to Lebanon’s environmental, economic, and infrastructural constraints. In a country where up to 80 percent of dairy consumption relies on imports, and where domestic production faces high input costs, political instability, and fragmented infrastructure, these traditional dairy foods provide a robust, locally adapted response. They embody knowledge systems that enable milk surpluses, including cow, sheep, and goat milk, to be converted into long-lasting, transportable, and nutritionally dense reserves that enhance food security where cold chains may fail or remain economically inaccessible.

Products such as Kishk, Shanklish, and Matured Labneh Balls demonstrate preservation strategies that allow for long-term storage without refrigeration, mitigating post-harvest losses and smoothing seasonal production cycles. Cheeses like Anbaris and its ball form illustrate the use of natural fermentation and salting to extend shelf life, while Karishi and Pressed–Brined Karishi (Lebanese Double-Cream) exemplify circular economy models through effective whey valorization. Even highly perishable products, such as fresh Labneh, fresh Halloumi, and Qishta, maintain critical roles within daily consumption patterns, especially where short supply chains and small-scale production ensure rapid turnover and freshness.

These insights suggest that efforts to align Lebanese dairy production with international classification systems should be pursued not only for export readiness and quality certification but also to support rural economies, promote food sovereignty, and preserve biocultural knowledge. Strategic hybridization, combining artisanal methods with selected industrial practices such as pasteurization, ultrafiltration, and standardized fermentation, can help producers achieve food safety, efficiency, and market integration while maintaining the distinctive sensory and cultural attributes that define Lebanese dairy heritage. Moving forward, targeted innovation must be grounded in context-specific strategies such as the development of rural micro-dairies, low-cost hygiene upgrades, modular preservation systems, and the operationalization of PGI frameworks through cooperative support and environmental compliance tools. These approaches offer a realistic and culturally appropriate path to strengthening economic resilience while safeguarding traditional know-how.

Ultimately, Lebanon’s traditional dairy sector represents not only a repository of historical culinary practices but also a strategic asset for building a more self-reliant, sustainable, and culturally resilient food system. Investing in its preservation, modernization, and promotion can strengthen economic resilience, support rural livelihoods, and ensure that this rich gastronomic heritage continues to contribute meaningfully to both local and international markets.

## Figures and Tables

**Table 1:** Cheese classification adapted from the Tetra Pak Dairy Processing Handbook [53].

Moisture on Fat-Free Basis (MFFB %) *	The First Phrase in the Designation Shall Be Term I	Fat in Dry Substance (FDS %) **	The Second Phrase in the Designation Shall Be Term II	Designation According to Principal Curing Characteristics Term III ^a^
<41%	Extra-hard	>60%	High-fat	❖ Status 1: Cured or ripened. (a) Mainly surface-ripened. (b) Mainly interior-ripened. ❖ Status 2: Mold-cured or ripened. (a) Mainly surface-ripened. (b) Mainly interior-ripened. ❖ Status 3 ***: Uncured or unripened fresh cheese.
49–56%	Hard	45–60%	Full-fat	
54–63%	Semi-hard	25–45%	Medium-fat	
61–69%	Semi-soft	10–25%	Low-fat	
>67%	Soft	<10%	Skim	

*: MFFB defines texture; MFFB = {Weight of moisture in cheese/{Total cheese weight − Weight of Fat in cheese}}. Note 1: Moisture ranges overlap to reflect typical variation in cheese processing and texture; these categories are guidelines rather than absolute boundaries. **: FDS indicates fat content when moisture is excluded; FDS = {Weight of fat in cheese/{Total cheese weight − Weight of Fat in cheese}}. ^a^: Ripening status (Term III) classifies cheese as: status 1: cured, status 2: mold-ripened, or status 3: fresh based on microbiological treatment and storage. ***: Milk intended for this cheese type should be pasteurized.

**Table 2:** Nutritional composition of key Lebanese traditional dairy products.

Product	Fat (%)	Protein (%)	Moisture (%)	Salt (%)	pH	Acidity (% LA *)	Notes
Fresh Labneh	~9.4	~7.7	~72.5	0–1	4.2	~1.02	Soft, spreadable, fresh/unripened
Labneh Balls	14–22	~11–12	~50–60	2–3	4.0–4.3	~1.5	Oil-preserved, reduced moisture
Labneh Anbaris	15–21	~6–9	45–70	3–4	~3.5	1.6–2.8	Fermented, stored in jars
Labneh Anbaris Balls	17–21	~9–12	45–55	3–4	~3.5	1.6–2.8	Dried or preserved in oil
Fresh Shanklish	19–21	~18–21	50–55	4–6	4.8–5.2	~1.2	Salted curd, mold-coated during ripening
Mature Shanklish	19–25	~19–21	45–50	4–6	4.5–5.0	~1.5	Mold-ripened, aged 2–6 weeks
Fresh Halloumi	23–25	~18–20	45–50	2–3	6.3–6.5	~0.5	High-moisture, fresh-brined
Brined (Mature) Halloumi	23–25	~18–20	40–45	5–7	5.8–6.2	~0.6	Brined for extended storage
Karishi (Lebanese Ricotta)	~10	~11–12	72–76	1–2	~6.2	~0.4	Fresh, unripened whey cheese
Pressed–Brined Karishi/Lebanese Double-Cream	12–15	~17	65–68	~4	~5.9	~0.5	Same product, local naming difference
Fresh Kishk	4–6	8–10	~25	1–2	~4.2	1.3–1.4	Fermented yogurt–bulgur paste
Dried Kishk	13–14	23–25	2.9–3.5	3–4	≤3.95	≥4.5	Shelf-stable, fermented powder

*: LA = lactic acid. Note 1: Pressed–Brined Karishi and Lebanese Double-Cream are the same product. Note 2: Data synthesized from different publications [3,6,7,14,16,18,19,23,26,32,38,40,41,43,44,45,50,62,63,66,71,72,73,78,79,80,85,86,87,88].

**Table 3:** Milk-to-product conversion values and classification.

Product	Kg Milk or Whey per kg Product	Classification (Codex/Tetra Pak)
Fresh Labneh	~2.6–3.0 milk	Unripened Fresh Cheese, Soft
Labneh Balls (Makbouseh)	~3.6–5.5 milk	Unripened Fresh Cheese, Lower-Moisture
Labneh Anbaris	~4 milk	Unripened Fermented Fresh Cheese, Soft
Labneh Anbaris Balls	~5.5 milk	Unripened Fermented Fresh Cheese, Lower-Moisture
Fresh Shanklish	~8.5–10 milk	Mold-Ripened Cheese, Semi-Soft
Mature Shanklish	~14 milk	Mold-Ripened Cheese, Semi-Hard
Fresh Halloumi	~8.5 milk	Semi-Hard Pasta Filata Style (Fresh)
Brined (Mature) Halloumi	~11 milk	Semi-Hard Cheese, Brined
Karishi (Lebanese Ricotta)	~20 whey	Whey Cheese (Acid–Heat-Coagulated), Fresh
Pressed–Brined Karishi/Lebanese Double-Cream	~40 whey	Whey Cheese (Pressed/Salted), Fresh
Fresh Kishk	~3.2 kg yogurt + 0.8 kg bulgur to give 4 kg	Fermented Dairy–Cereal Composite, High-Moisture
Dried Kishk	Input of fresh Kishk yields ~1 kg	Fermented Dairy–Cereal Powder, Shelf-Stable

Note 1: Pressed–Brined Karishi and Lebanese Double-Cream are the same product. Note 2: classification categories are based on Table 1 [53,61]. Note 3: Conversion ratios are compiled from different publications [3,6,7,14,16,18,19,23,26,32,38,40,41,43,44,45,50,62,63,66,71,72,73,78,79,80,85,86,87,88].

**Table 4:** Industrial vs. traditional production: yield, labor, and processing time.

Product	Production Mode	Milk Required (kg per kg Product)	Time to Final Product
Fresh Labneh	Traditional	~3.0–3.5	Overnight
	Industrial	~2.5–3.0	6–12 h
Labneh Balls	Traditional	~3.6–5.5	2–5 days straining, balling, add oil
	Industrial	Not yet industrialized	Not available
Labneh Anbaris	Traditional	~4.5–5.3	Variable, until clay jar is filled
	Industrial	~4.5–5.3	3 days (Batch wise)
Labneh Anbaris Balls	Traditional	~5.5	2–5 days straining, balling, add oil
	Industrial	Not yet industrialized	Not available
Fresh Shanklish	Traditional	~8.5–10	2–6 weeks aging
	Industrial	Not yet industrialized	Not available
Mature Shanklish	Traditional	~14.0	Up to 12 months aging
	Industrial	Not yet industrialized	Not available
Fresh Akkawi	Both	~6	1–2 days including brining
Mature Akkawi “Tsheeky”	Both	~7.5	Several weeks to months in brine
Fresh Halloumi	Both	~8.5	1–3 days including brining
Brined (Mature) Halloumi	Both	~11	Several weeks to months in brine
Karishi (Lebanese Ricotta)	Home-scale	Limited whey availability	Household production limited by whey access
	Industrial	From whey (approx. 10 L milk → 9 L whey → 0.4 kg)	1–2 h heating and draining
Pressed–Brined-Karishi/Lebanese Double-Cream	Home-scale	Limited whey availability	Household production limited by whey access
	Industrial	From whey (approx. 20 L milk → 9 L whey → 0.5 kg)	2–3 h with pressing and brining for few days
Qishta	Both	~3.5–4.0	2–3 h gentle heating and skimming
Fresh Kishk	Traditional	~3.2 yogurt + 0.8 bulgur	Several days fermentation, optional oil storage
	Industrial	Not yet industrialized	Not available
Dried Kishk	Traditional	~5.0 (milk equivalent) + bulgur	Weeks fermentation plus sun-drying
	Industrial	Not yet industrialized	Not available

Note 1: Pressed–Brined Karishi and Lebanese Double-Cream are the same product. Note 2: Estimated processing times, yield ratios, and production modes are synthesized from published studies and reported artisanal practices [3,6,7,14,16,18,19,23,26,32,38,40,41,43,44,45,50,63,66,71,72,73,78,79,80,85,86,87,88].

**Table 5:** Shelf life, refrigeration requirements, preservation method, and predicted water activity of traditional Lebanese dairy products (shelf life estimated).

Product	Shelf Life	Refrigeration	Preservation Method	Predicted aw Category
Fresh Labneh	1–2 weeks	Yes	Refrigerated in sealed container with acidification	>0.90 (high)
Labneh Balls	1–6 months	No (if sealed)	Olive oil storage maintaining low water activity	~0.85 (moderate)
Labneh Anbaris	3–6 months	No	Natural brine in clay jars with salt and fermentation	~0.85 (moderate)
Shanklish	3–12 months	No	Airtight aging with drying and mold ripening	<0.85 (moderate–low)
Fresh Halloumi	2–4 weeks	Yes	Brining under refrigeration	~0.85 (moderate)
Brined (Mature) Halloumi	Up to 6 months	Not always	Brined and vacuum-packed	~0.85 (moderate)
Fresh Akkawi	2–4 weeks	Yes	Brining under refrigeration	~0.85 (moderate)
Mature Akkawi (Akkawi Tscheeky)	Up to 6 months	Not always	Brined and vacuum-packed	~0.85 (moderate)
Karishi (Lebanese Ricotta)	3–5 days	Yes	Refrigerated container with acidification	>0.90 (high)
Pressed–Brined Karishi/Lebanese Double-Cream	1–3 weeks	Yes	Brined or vacuum-packed for added density	~0.85 (moderate)
Qishta	2–3 days	Yes	Gentle heating and skimming of milk skin	>0.90 (high)

Note 1: Pressed–Brined Karishi and Lebanese Double-Cream are the same product. Note 2: Shelf life estimates and preservation methods are derived from artisanal practice and published studies, including [3,6,16,18,19,23,24,26,38,40,41,43,44,45,50,62,63,66,71,72,79,88]. Note 3: Predicted water activity (aw) categories are indicative, based on typical moisture and salt content and empirical models for aw prediction [93]. General thresholds of aw < 0.85 inhibit bacterial spoilage, while aw < 0.70 further prevents fungal and mold growth [94].

## Data Availability

No new data were created or analyzed in this study. Data sharing is not applicable to this article.
